# HAP2(GCS1)-Dependent Gamete Fusion Requires a Positively Charged Carboxy-Terminal Domain

**DOI:** 10.1371/journal.pgen.1000882

**Published:** 2010-03-19

**Authors:** Julian L. Wong, Alexander R. Leydon, Mark A. Johnson

**Affiliations:** Department of Molecular Biology, Cell Biology, and Biochemistry, Brown University, Providence, Rhode Island, United States of America; Stanford University School of Medicine, United States of America

## Abstract

HAP2(GCS1) is a deeply conserved sperm protein that is essential for gamete fusion. Here we use complementation assays to define major functional regions of the *Arabidopsis thaliana* ortholog using HAP2(GCS1) variants with modifications to regions amino(N) and carboxy(C) to its single transmembrane domain. These quantitative in vivo complementation studies show that the N-terminal region tolerates exchange with a closely related sequence, but not with a more distantly related plant sequence. In contrast, a distantly related C-terminus is functional in Arabidopsis, indicating that the primary sequence of the C-terminus is not critical. However, mutations that neutralized the charge of the C-terminus impair HAP2(GCS1)-dependent gamete fusion. Our results provide data identifying the essential functional features of this highly conserved sperm fusion protein. They suggest that the N-terminus functions by interacting with female gamete-expressed proteins and that the positively charged C-terminus may function through electrostatic interactions with the sperm plasma membrane.

## Author Summary

Recent studies suggest that HAP2(GCS1) is a deeply conserved protein required for gamete membrane fusion, a critical yet poorly understood step in sexual reproduction. HAP2(GCS1) is present in many plant, protist, and animal genomes, and has been shown to be essential for fertilization in Arabidopsis, Chlamydomonas, and Plasmodium. The loss-of-function phenotype in Chlamydomonas suggests a direct role in gamete plasma membrane fusion. HAP2(GCS1) has no known functional domains, making it difficult to predict how it contributes to gamete fusion. We set out to map the critical features of this protein by testing a series of deletions, substitutions, and interspecific chimeras for their ability to rescue the *hap2-1* fertilization defect in Arabidopsis. We found that the N-terminus does not tolerate sequence divergence, but the histidine-rich C-terminus does. We propose that the N-terminus of HAP2(GCS1) functions in part by interacting with proteins on the surface of female gametes. The key feature of the C-terminus is positive charge, a characteristic that could favor interactions with the plasma membrane that promote membrane fusion. Our studies provide a description of HAP2(GCS1) functional domains and provide an important framework for defining the role of this essential component of a conserved reproductive mechanism.

## Introduction

The fusion of gamete plasma membranes is a critical event in fertilization, but despite the ubiquity of the process among sexually reproducing eukaryotes, no conserved mechanism for gamete fusion has been described. At least two factors contribute to our lack of mechanistic insight. First, many proteins that mediate binding and fusion of complementary gametes evolve rapidly, thereby reinforcing barriers to interspecific hybridization [1]. Second, gamete fusion is a transient event occurring between two cells, limiting the ability to observe fusion and to study it using biochemical methods.

Genetic analysis in Arabidopsis (*Arabidopsis thaliana*, At) identified *HAP2(GCS1)*, a sperm-expressed gene that is essential for fertilization [2]–[4]. In flowering plants, two genetically identical haploid sperm are delivered by a pollen tube to female gametes that develop within an ovule. One sperm fuses with the egg to produce a zygote while the other fuses with the central cell to produce endosperm, a tissue that supports the developing embryo. Both fertilization events are required to initiate development of a seed (reviewed in [5]–[6]). *HAP2(GCS1)*, for *HAPLESS2* [2],[4] and synonym *GENERATIVE CELL SPECIFIC1* [3], is required for both sperm fusion events occurring during double fertilization.

The role of *HAP2(GCS1)* in fertilization may be widespread in eukaryotes as orthologs are present in several protist, animal, and plant genomes [7]–[8]. Loss of *HAP2(GCS1)* function in male gametes also blocks fertilization in *Plasmodium berghei* (sperm affected [7],[9]) and *Chlamydomonas reinhardtii* (Cr, *minus* gametes affected [7]), suggesting it plays a similar role at fertilization throughout eukaryotes. Key observations made in Chlamydomonas suggest a specific role for CrHAP2(GCS1) in gamete fusion [7]: (i) The *Cr hap2(gcs1)* loss-of-function mutation prevents fertilization, despite the ability of gametes to bind one another and bring opposing membranes into close proximity and (ii) CrHAP2(GCS1) is enriched at the tip of the *minus* mating projection just prior to fusion.

**Figure 1:**
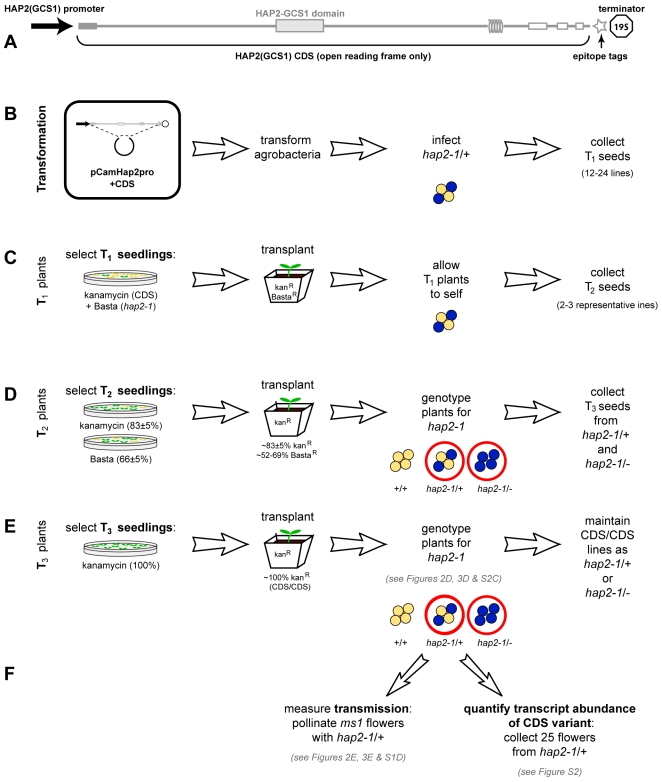
Protocol for analyzing complementation of *hap2-1* fertilization defects using *HAP2(GCS1)* CDS variants. (A) Schematic of the AtN•AtC construct representing the 1.5 kb promoter, *HAP2(GCS1)* coding sequence with major domains, C-terminal V5C4 epitope tag, and 19S terminator and polyadenlyation sequence. Grey box represents the predicted signal sequence in exon 1, coils represent the predicted transmembrane domain. The HAP2-GCS1 domain (Protein Family ID: PF10699; InterPro ID, IPR018928) is the most conserved sequence among orthologs. (B–F) Method to obtain single-locus-insertion transgenic plants for complementation analysis. (B) *hap2-1/+* plants were transformed. (C) Primary transformants (T_1_) plants were selected for both Basta^R^ and kan^R^, indicating they carried *hap2-1* and the CDS construct. (D) The progeny (T_2_) of self-fertilization of these individuals (T_1_) were assayed for segregation of the CDS construct and *hap2-1* by scoring kan^R^ and Basta^R^. Self-fertilization of *hap2-1* resulted in 50% Basta^R^ progeny [2],[4]. Complementation of the *hap2-1* transmission defect by a single locus insertion of a CDS encoding functional HAP2(GCS1) increases this to ∼66% Basta^R^ in the progeny of T_1_ plants. We screened progeny from 15–24 transformants and selected at least two lines whose progeny exhibited ∼66% Basta^R^. In cases where complementation failed and no lines resulted in >50% Basta^R^ progeny, we chose two lines siring the highest percentage Basta^R^ progeny for further analysis. We identified and selected transgenic lines with a single insertion of the CDS construct by analyzing kan^R^ among progeny. (E) Lines homozygous for a CDS variant (CDS/CDS) were identified by scoring kan^R^ in T_3_ progeny (100%). (F) Assays used to evaluate expression and complementation of each *hap2-1/+,* CDS/CDS line. The descendents of at least two individuals from each transgenic line were used to conduct each assay.

**Figure 2:**
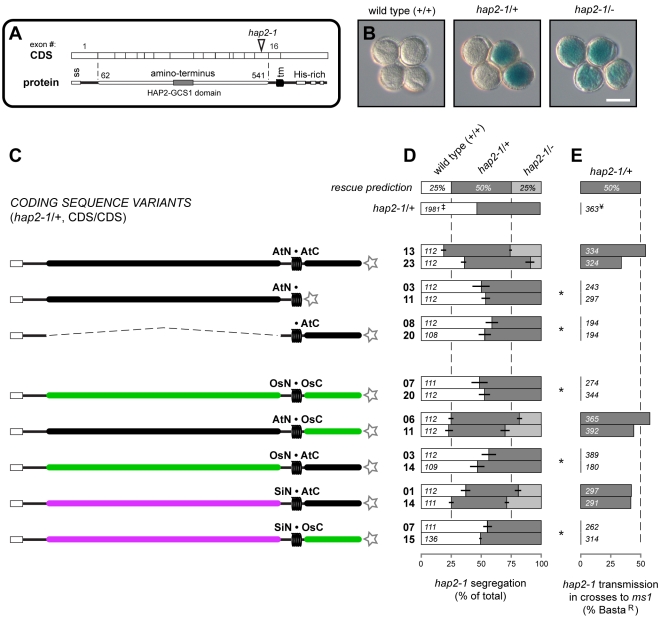
Analysis of *HAP2(GCS1)* deletions and interspecific chimeras. (A) Schematic of the HAP2(GCS1) CDS and protein. Exon boundaries (numbered above) are mapped to the open reading frame and major features including the signal sequence (ss), transmembrane domain (tm), Arabidopsis amino acid position numbers delineating the N-terminal region, and the T-DNA insertion for *hap2-1* are shown. (B) Pollen tetrads from wild-type (+/+), *hap2-1/+,* or *hap2-1/-* plants. Scale bar = 50 µm. (C) Diagram of the HAP2(GCS1) variants tested. Star at the C- terminus represents the epitope tag. Genbank accession numbers: *A. thaliana*  =  DQ022375; *S. irio*  =  GU724984; *O. sativa*  =  AK072871. (D-E) Percentage of progeny inheriting *hap2-1* from *hap2-1/+*, CDS/CDS plants following self-fertilization (D) or crosses of pollen to *ms1* pistils (E). Expected frequency of progeny inheriting *hap2-1* if variant is fully functional is shown in the top bar (“rescue prediction”), and previously published *hap2-1* results are shown for comparison (‡, [2] and ¥, [4]). Data from two independent transgenic lines are shown for each HAP2(GCS1) variant. Bars represent the mean percentages from each transgenic line; horizontal lines denote the percentage range between descendents from two individuals per line. Total number of plants (D) or seedlings (E) scored per line is shown. Asterisks denote significant differences from expected complementation (*p*<10^−5^).

All predicted HAP2(GCS1) orthologs share a common primary architecture. Each is divided into two regions by a single pass transmembrane domain and contains a HAP2-GCS1 domain of about 50 amino acids in the large region amino(N)-terminal to the transmembrane domain (Figure 1A and Figure 2A). The carboxy(C)-terminus is enriched in charged residues that do not follow a defined sequence: histidine is dominant in flowering plants while other basic residues (lysine, arginine) are enriched in other species [3]–[4], [7]–[8]. Primary sequence analysis of HAP2(GCS1) has not detected other known motifs or functional domains.

We use quantitative molecular-genetic assays in Arabidopsis to characterize the major features of HAP2(GCS1). We find that both regions of the protein are essential for function, and that these regions are under different selective pressures: Primary sequence from a closely related plant, not a distant relative, can replace the Arabidopsis N-terminus. Thus, while HAP2(GCS1) does not define species-level interactions between gametes, function of the N-terminus may be constrained by co-evolution with partner proteins expressed by female gametes. On the other hand, the Arabidopsis HAP2(GCS1) C-terminus retains function when replaced with sequence from a distantly related plant or mutated sequences, as long as positive charge is retained. Thus, net charge over the C-terminus is the critical feature of this region. These experiments thus establish essential characteristics of an ancient protein required for gamete fusion.

## Results

### A Genetic Assay to Evaluate the In Vivo Function of HAP2(GCS1) Variants

*hap2-1* blocks the ability of sperm to participate in fertilization, but does not affect female reproduction [4]. This allele was generated by insertion of a T-DNA carrying two marker genes to facilitate analysis of segregation and transmission: (i) resistance to the herbicide Basta (Basta^R^) and (ii) β-glucuronidase (GUS) driven by the pollen-specific LAT52 promoter (*LAT52:GUS*) [2],[4]. *hap2-1* was identified in the *quartet (qrt1-2)* background, a mutation that maintains male meiotic products in tetrads [10]. This feature, combined with *LAT52:GUS* expression in pollen, allows one to distinguish heterozygous *hap2-1* (*hap2-1/+*) pollen from wild-type (Figure 1B–1F and Figure 2B). Self-fertilization of *hap2-1/+* results in 50% heterozygous (Basta^R^, two GUS^+^ to two GUS^-^ pollen per tetrad) and 50% wild-type plants (Basta sensitive (Basta^S^), GUS^-^ pollen); homozygous *hap2-1 (hap2-1/-)* plants are not recovered [2],[4], and thus *hap2-1* transmission is distorted (not the expected 1∶2∶1 segregation of wild type: heterozygous: homozygous mutant). Furthermore, pollination of wild-type females with *hap2-1/+* pollen yields no progeny with the *hap2-1* allele, e.g. all progeny are sired by wild-type pollen produced by the heterozygous father. Thus, *hap2-1* cannot be transmitted though the male germline.

We developed a system to test if coding sequence (CDS) variants of *HAP2(GCS1)* could restore male transmission of the *hap2-1* allele (Figure 1). Transformation of *hap2-1/+* mutants with a wild-type *HAP2(GCS1)* genomic clone [including 1.5 kb of *HAP2(GCS1)* promoter sequence] complemented the fertilization defect, and self-fertilization of *hap2-1*/+ mutants carrying this transgene produced *hap2-1/-* progeny (Basta^R^, four GUS^+^ pollen per tetrad) [4]. Pollen from these plants was also capable of transmitting *hap2-1* to progeny when crossed to wild-type females, producing Basta^R^ *hap2-1/+* seedlings [4]. We transformed *hap2-1/+* plants with a series of *HAP2(GCS1)* CDS variants under the control of the same 1.5 kb *HAP2(GCS1)* promoter sequence; each variant T-DNA construct carried a kanamycin resistance (Kan^R^) gene. To track expression of CDS constructs and to differentiate between endogenous and introduced *HAP2(GCS1)*, we included sequences encoding short epitope tags (V5 [11] and tetra-cysteine (CCGPCC) [12]; see Materials and Methods) at the 3′ end of the variants (Figure 1A).

To determine if *HAP2(GCS1)* variants were capable of mediating gamete fusion, we generated *hap2-1/+* transgenic lines that were homozygous for the CDS variant (CDS/CDS, Figure 1). These plants produce two pollen genotypes whose ability to fertilize female gametes could be directly compared: (i) *HAP2(GCS1)*, CDS (Basta^S^, GUS^-^, Kan^R^) or (ii) *hap2-1*, CDS (Basta^R^, GUS^+^, Kan^R^). As with the genomic construct [4], if the CDS variant encodes fully functional HAP2(GCS1), the ability to fertilize wild-type females, and thus transmit *hap2-1*, should be restored to *hap2-1* sperm.

Introduction of either the native CDS (data not shown), or an epitope tagged version rescued fertility of *hap2-1* (Figure 2C–2E, top row, and Figure S1). In both transgenic lines analyzed, *hap2-1/-* plants were recovered following self-fertilization, and segregation was restored to ∼1∶2∶1 (25% wild-type, 50% *hap2-1/+*, 25% *hap2-1/-*, Figure 2D). When these lines were used to pollinate *male sterile1* (*ms1*) females, *hap2-1* was inherited (Figure 2E and Figure S1), indicating complete or nearly complete complementation of *hap2-1* by the epitope-tagged, native Arabidopsis *HAP2(GCS1)* CDS. These control experiments demonstrated that the addition of C-terminal epitope tags did not disrupt the function of the *HAP2(GCS1)* CDS and that expressing the *HAP2(GCS1)* CDS from the *HAP2(GCS1)* promoter resulted in expression of functional HAP2(GCS1) protein.

### Regional HAP2(GCS1) Deletions Are Not Functional

We first asked if the regions N- or C-terminal to the HAP2(GCS1) transmembrane domain were essential for HAP2(GCS1) function. The amino acids encoded by exons 2-15 (amino acid residues 62–541, Figure 2A) were deleted, retaining exon 1 and its signal peptide to ensure that the protein product was properly directed to the secretory pathway (•AtC, Figure 2C). In a second construct, we directly fused epitope tags to the end of the transmembrane domain to test if the C-terminus was essential (AtN•, Figure 2C). None of the 24 primary transformants established for either variant segregated >50% Basta^R^ seedlings, produced *hap2-1/-* plants, or restored normal segregation among the progeny of self-fertilization (Figure 2D and Figure S1). Further, *hap2-1*/+ pollen did not produce Basta^R^ progeny when crossed to *ms1* (Figure 2E and Figure S1).

Epitope tag sequences were detected in floral mRNA extracted from AtN• or •AtC lines, and the abundance of *HAP2(GCS1)* mRNA was higher in these flowers than in *hap2-1/+* flowers (Figure S2), suggesting that failure of •AtC or AtN• variants to rescue *hap2-1* was not due to lack of construct expression. Thus, the two regions of HAP2(GCS1) that lie on either side of the transmembrane domain are essential for function.

### Exchange of the Arabidopsis Amino Terminus with Sequence from a Closely Related Species Produces a Functional Protein

We next asked if replacement of the major regions of Arabidopsis HAP2(GCS1) with sequences from plant orthologs rescued *hap2-1*. We chose rice (*Oryza sativa*, Os) as a representative monocot sequence; monocots and dicots diverged at least 200 million years ago [13]. OsHAP2(GCS1) is 59% identical with Arabidopsis in the N-terminal region and 37% identical at the C-terminus [4] (Figure S3). Expression of the OsHAP2(GCS1) CDS from the AtHAP2(GCS1) promoter failed to rescue the Arabidopsis *hap2-1* fertilization defect (not shown). To ensure that the rice protein was properly expressed and localized in Arabidopsis sperm, we replaced Arabidopsis exons 2-15 and the Arabidopsis C-terminus with the orthologous rice sequences to maintain the Arabidopsis signal sequence and transmembrane domain (OsN•OsC, Figure 2C and Figure S1). This exchange also failed to rescue the *hap2-1* defect (Figure 2D and 2E and Figure S1).

In contrast, a chimera consisting of the Arabidopsis N-terminal region and the rice C-terminal region was fully functional (AtN•OsC, Figure 2C–2E). Surprisingly, the reciprocal variant made by exchanging Arabidopsis exons 2–15 with the more conserved rice N-terminal sequence, was not functional (OsN•AtC, Figure 2C–2E and Figure S1). However, a similar chimera made with sequence from *Sisymbrium irio* (Sisymbrium, Si, 89% identical N-terminus, Figure S3), a closely related member of the same family as Arabidopsis (Brassicaceae [14]), did complement *hap2-1* (SiN•AtC, Figure 2C–2E and Figure S1). This result suggests that the failure of OsN•AtC to rescue *hap2-1* was a consequence of primary sequence divergence. Thus, conservation of primary amino acid sequence is essential for proper function of the N-terminus, but not the C-terminus. A Sisymbrium N-terminus / rice C-terminus chimera was not functional (SiN•OsC, Figure 2C–2E and Figure S1) even though each of these regions can function when paired with the complementary Arabidopsis sequence. This result suggests that the Sisymbrium N-terminus and the rice C-terminus have reduced function compared to their Arabidopsis counterparts, and that this hybrid CDS produces a non-functional protein.

### Positive Charge Is the Principal Functional Feature of the HAP2(GCS1) C-Terminus

The ability of the AtN•OsC chimera to rescue *hap2-1* implies that the greater sequence diversity in the C-terminus compared to the N-terminus [3]–[4],[7] may be a consequence of evolutionary drift rather than the influence of positive selection, which can also produce such primary sequence diversity [1]. The drift hypothesis is further supported by the observation that the C-terminal enrichment in histidine residues is so far limited to flowering plants [3]–[4], [7]–[8]. Thus, localized positive charge at the C-terminus of HAP2(GCS1) may be functionally more important than primary sequence.

**Figure 3:**
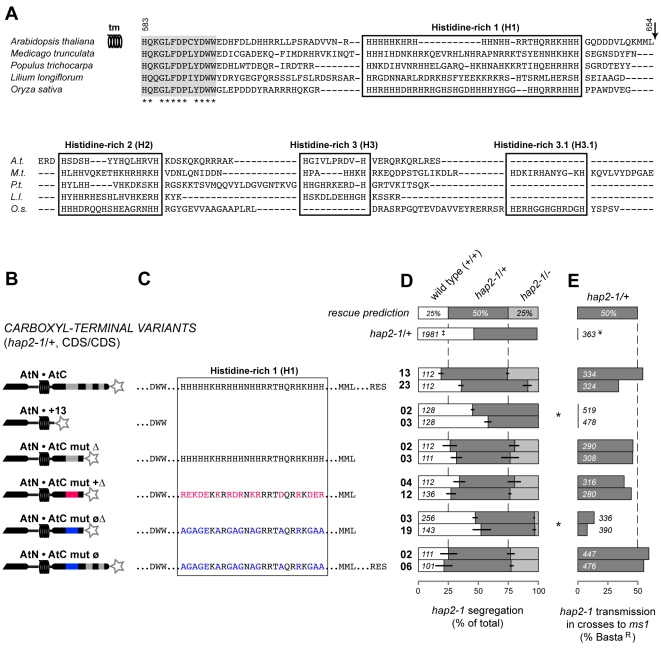
Positive charge in the C-terminal region is required for HAP2(GCS1) function. (A) Alignment of HAP2(GCS1) C-termini from selected angiosperms for which complete coding sequences are available (Genbank accessions dicots: *A. thaliana*  =  DQ022375, *M. trunculata*  =  AC146573, *P. trichocarpa*  =  XP_002298057; monocots: *L. longiflorum*  =  BAE71142, *O. sativa*  =  AK072871). Thirteen conserved amino acids after the transmembrane domain (grey) and histidine-rich domains (boxed) are highlighted. Arrow marks the point where a partial C-terminal truncation was made (AtN•AtC mut Δ, and variants). Numbers correspond to key residues in the Arabidopsis primary sequence. (B) Diagram of the C-terminal HAP2(GCS1) variants tested. Star at the C-terminus of the diagrams represents the epitope tag. (C) Sequences of the H1 domain in each C-terminal variant. (D–E) are presented as in Figure 2.

We dissected the Arabidopsis HAP2(GCS1) C-terminus to determine what features were required for function. Alignments of flowering plant C-termini representing dicots and monocots revealed a run of 13 conserved amino acids immediately after the predicted transmembrane domain followed by three histidine-rich domains. The longest histidine-rich stretch is adjacent to the transmembrane domain, and has an average *pI* of 12.5 (Figure 3A). A variant composed of the complete amino terminus plus the conserved 13 amino acids was not functional (AtN•+13, Figure 3B–3E and Figure S1). However, extending the C-terminus to include the first histidine-rich domain (H1) resulted in a fully functional variant (AtN•AtC mut Δ, Figure 3D and 3E and Figure S1). Replacement of all histidines in this truncated version with other polar, charged amino acids (arginine and lysine) also resulted in a variant that complemented *hap2-1* (AtN•AtC mut +Δ, *pI* = 11.4, Figure 3B–3E and Figure S1). In contrast, replacing histidines with nonpolar residues (glycine and alanine) significantly impaired the function of HAP2(GCS1) (AtN•AtC mut øΔ, *pI* = 12.2, Figure 3B–3E and Figure S1). When this neutralized domain was extended to include the downstream histidine-rich domains H2 and H3, function was restored (AtN•AtC mut ø, *pI* = 11.1, the same as the endogenous sequence; Figure 3B–3E and Figure S1). Thus, a hypomorph of *HAP2(GCS1)* can be made by neutralizing the C-terminus with nonpolar residues.

We observed the following trend among the C-terminal variants tested. Complete removal of the C-terminus (AtN•) or histidine-rich region (AtN•+13) abrogates HAP2(GCS1) function. C-termini consisting of the endogenous H1 or positively charged H1 variants are fully functional. However, substituting nonpolar residues for histidine in H1 domain generates a minimally functional protein, yielding only 2–4% *hap2-1/-* progeny from self-fertilization and 9–15% transmission of the *hap2-1* allele in crosses to *ms1* females (Figure 3E and Figure S1).

### The AtN•AtC mut øΔ Hypomorph Disrupts Sperm Function in Fertilization

**Figure 4:**
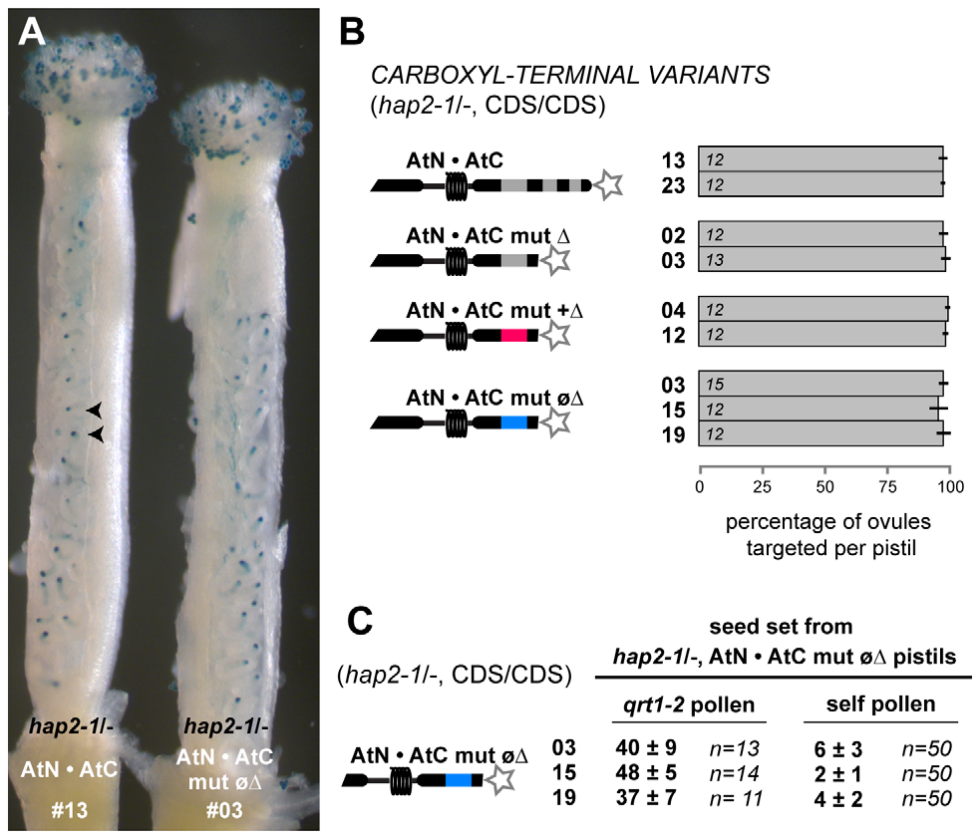
AtN•AtC mut øΔ is a hypomorph of HAP2(GCS1) that affects only male fertility. (A) Representative pistils of self-pollinated plants homozygous for *hap2-1* and the *HAP2(GCS1)* variant (*hap2-1*/-, CDS/CDS). Ovules targeted by pollen tubes are indicated by the blue dots (GUS staining, arrowheads). Image shows pistils from *hap2-1/-*, AtN•AtC line #13 (left) and *hap2-1/-*, AtN•AtC mut øΔ #03 (right). (B) Percentage of ovules targeted per pistil. Number of pistils scored per line is shown. (C) Seed set per silique when *hap2-1/-*, AtN•AtC mut øΔ pistils are crossed with *qrt1-2* pollen (left) or manually self-pollinated (right). Number of siliques scored is indicated.

We further characterized rare AtN•AtC mut øΔ, *hap2-1/-* plants to understand the effect of the nonpolar C-terminus on HAP2(GCS1) function. Two sperm were present in AtN•AtC mut øΔ, *hap2-1* pollen grains (97–99%, *n>500* per line), indicating gametophyte development was normal. Furthermore, AtN•AtC mut øΔ, *hap2-1* pollen tubes were able to target ovules and deliver sperm, as judged by counting the number of ovules that received LAT52:GUS activity 7.5 hours after manual self-pollination (Figure 4A and 4B). Female function was not affected in AtN•AtC mut øΔ, *hap2-1/-* lines, and full seed-set was obtained when pistils were pollinated with *qrt1-2* pollen (Figure 4C). However, when AtN•AtC mut øΔ, *hap2-1/-* plants were allowed to self-fertilize, only four to seven seeds formed in each silique (∼10%, assuming an average of 50 seeds per normal silique; Figure 4C).

**Figure 5:**
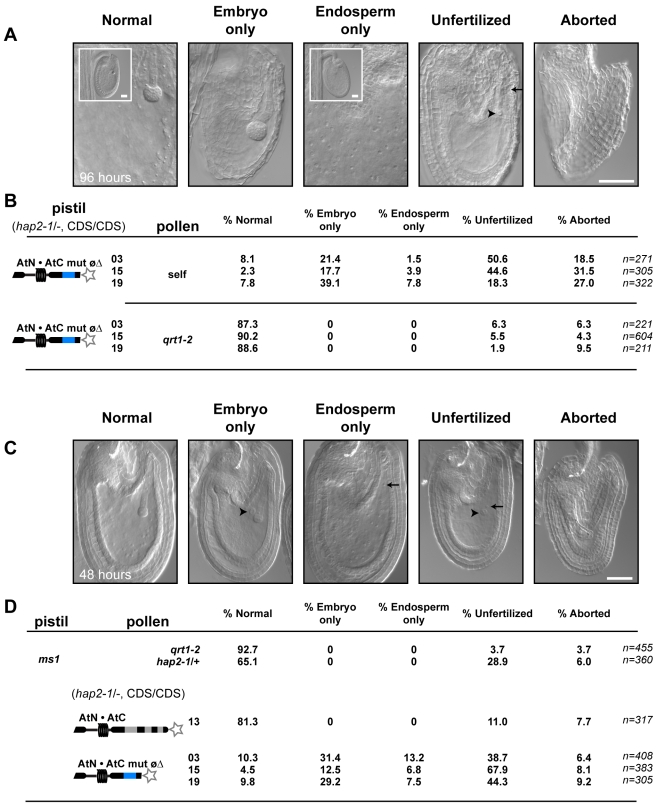
AtN•AtC mut øΔ, *hap2-1* sperm are defective at double fertilization. (A) Examples of the products of fertilization by *hap2-1*, AtN•AtC mut øΔ sperm 96 hours after self-pollination. Scale bar = 50 µm. (B) Frequency of observed fertilization events 96-hours after pollination with self (top) or *qrt1-2* (bottom) pollen. Number of ovules scored is indicated. (C) Examples of *ms1* ovules fertilized by *hap2-1, AtN•AtC* mut øΔ sperm 48 hours after pollination. (D) Frequency of observed fertilization events 48-hours after pollination of *ms1* pistils with pollen from the indicated line. Number of ovules scored is indicated.

The finding that AtN•AtC mut øΔ, *hap2-1* sperm were being released in nearly all ovules, yet seed formation was dramatically reduced, suggested that the *HAP2(GCS1)* hypomorph specifically disrupted fertilization. Analysis of embryo and endosperm development four days after self-pollination of AtN•AtC mut øΔ, *hap2-1/-* plants revealed normal development in 2–8% of ovules (Figure 5A and 5B), consistent with the number of normal seeds observed after self-fertilization (Figure 4C). We also found that 18–50% of ovules remained unfertilized (Figure 5A and 5B), consistent with lack of HAP2(GCS1) function. In addition, we observed a significant number of ovules that contained either an embryo or endosperm, but not both products of double fertilization. Single fertilization events were not observed when *qrt1-2* pollen was used to pollinate AtN•AtC mut øΔ, *hap2-1/-* pistils (Figure 5B). Analysis of ovule development in *ms1* pistils pollinated with AtN•AtC mut øΔ, *hap2-1* pollen two days after pollination also yielded significant numbers of unfertilized and singly fertilized ovules (Figure 5C and 5D). When *ms1* pistils were pollinated with *qrt1-2*, *hap2-1/+*, or *hap2-1/-* carrying the functional AtN•AtC CDS, however, no single fertilization events were observed. About 25% of the ovules in pistils pollinated with *hap2-1/+* pollen remain unfertilized (Figure 5D), as previously reported [4].

These data suggest that neutralizing HAP2(GCS1) C-terminal charge crippled function. In the majority of cases, neither sperm was capable of fusing with the egg or central cell (unfertilized, Figure 5). However, in a significant number of ovules, HAP2(GCS1) function dropped below a critical threshold in only one of the two sperm, producing single fertilization events (embryo-only, endosperm-only, Figure 5). These results are consistent with the conclusion that AtN•AtC mut øΔ represents a hypomorph of HAP2(GCS1).

## Discussion

In vivo analysis of HAP2(GCS1) variants has defined two regions that play distinct roles during HAP2(GSC1)-mediated double fertilization. The protein can be divided into N- and C-terminal regions based on the position of the transmembrane domain. Both regions are essential for function, but different evolutionary constraints are driving their roles in fertilization.

We propose that an extracellular orientation of the N-terminus allows this region to regulate gamete fusion by its interaction with factors on the egg or central cell. This organization is consistent with Hidden Markof Modeling (www.cbs.dtu.dk/services/TMHMM-2.0/); with the invariant conservation of cysteine residues within the HAP2-GCS1 domain that are predicted to participate in disulfide bonding in an extracellular environment; and with the successful use of N-terminal epitopes to produce antibodies that block Plasmodium reproduction [15]. The conserved HAP2-GCS1 domain could, for example, interact with another membrane-bound protein on female gametes facilitating the juxtaposition of the two plasma membranes. Our analysis of interspecific HAP2(GCS1) chimeras is consistent with an extracellular orientation of the N-terminus. Replacement of the Arabidopsis N-terminus with that of a closely related species (Sisymbrium, 89% identical) generated a HAP2(GCS1) variant capable of mediating fusion with Arabidopsis female gametes, but a variant generated with a distantly related sequence failed (rice, 59% identical). These data are consistent with the hypothesis that the egg and central cell express a protein that interacts with HAP2(GCS1) to mediate fusion, and that this protein:protein interaction fails beyond a certain level of sequence divergence in the HAP2(GCS1) N-terminal domain.

Our data also show that HAP2(GCS1) does not contribute to a species level barrier to hybridization. Wind and/or animals indiscriminately pollinate many flowering plant species, so it is important to consider mechanisms that limit hybridization. In some organisms, protein:protein interactions essential for complementary gamete binding and fusion are rapidly co-evolving to enhance reproductive isolation of one species from another [1],[16]. We observe that the N-terminus of Sisymbrium, but not rice HAP2(GCS1), can mediate fertilization with Arabidopsis female gametes. Arabidopsis and Sisymbrium are in the Brassica family, but belong to distinct tribes [14]. Thus, our data suggest that Arabidopsis female gametes can distinguish between the N-terminal sequences of HAP2(GCS1) from Arabidopsis and distantly related rice, but cannot discriminate Arabidopsis from closely related Sisymbrium. The recent finding that pollen tubes are attracted to ovules by small proteins with species-specific activity [17] supports a model that barriers prior to gamete-gamete interaction account for species-level discrimination in flowering plants, potentially leaving the proteins involved in gamete-gamete interactions to evolve without diversifying selection.

Positive charge, not primary amino acid sequence, is the C-terminal characteristic conserved among HAP2(GCS1) orthologs and our data show that positive charge is required for function. Unlike the protein:protein interactions proposed for the N-terminus, the intracellular C-terminus may be functioning through electrostatic interactions with negatively charged molecules (e.g. the inner face of the plasma membrane) that favor membrane fusion. Positively charged domains located on the intracellular domain of fusion-associated small transmembrane (FAST) proteins have been implicated in fusion of host cells by non-enveloped viruses [18].

Flowering plant C-termini are enriched in histidine whereas other positively charged amino acids (arginine and lysine) are prevalent in other orthologs [4], [7]–[8], suggesting that selection for one class of charged amino acids over another has shaped the evolution of HAP2(GCS1) in different eukaryotes. These three positively charged amino acids were functionally interchangeable in our Arabidopsis experiments. In nature, however, differences in the composition of the C-terminal domain may have been selected to meet the unique demands of the reproductive systems that use HAP2(GCS1). Under physiologic pH (e.g. pH 5–7), histidine exists in either a protonated or neutral form (pKa = 6.08) whereas lysine (pKa = 10.5) and arginine (pKa = 12.0) are always protonated. Perhaps the difference in sperm delivery mechanisms between flowering plants and other eukaryotes selected for the bimodal charge state of histidine. Flowering plant sperm develop within the pollen cytoplasm and are delivered to the ovule by a pollen tube. *hap2-1* pollen tubes have a reduced ability to target ovules compared to wild type, suggesting that in flowering plants, HAP2(GCS1) may have a role in pollen tube guidance that is distinct from its essential role in gamete fusion [4]. Future experiments will test these hypotheses by determining if HAP2(GCS1) variants with modified C-termini can complement the pollen tube guidance defect observed when *hap2-1* pollen tubes compete with wild-type pollen tubes for access to ovules. Pollen tubes burst upon arrival at the ovule, exposing sperm to the extracellular environment. It will be interesting to determine if this change in environment results in a drop in sperm pH that activates HAP2(GCS1) function.

*hap2-1* sperm expressing a HAP2(GCS1) variant with a neutralized C-terminus (AtN•AtC mut øΔ) had significantly reduced fertility. Double fertilization occurred in only ∼7% of the ovules we analyzed, while many ovules remained unfertilized (∼40%). A large portion of the ovules contained products of single fertilization events (∼23% embryo-only, 8% endosperm-only) that fail to complete seed development. These results highlight a unique advantage of flowering plants for the study of gamete fusion: the outcomes of two distinct fertilization events, both requiring HAP2(GCS1) function, can be observed independently. This situation provides a sensitive means to detect reduced fusion efficiency. We consistently observed fertilization of only one female gamete when two *hap2-1* sperm expressing the AtN•AtC mut øΔ HAP2(GCS1) variant were delivered to an ovule, specifically detecting more single fertilizations with the egg (embryo-only) than the central cell (endosperm-only). This suggests that sperm:central cell fusion may require more HAP2(GCS1) activity or that central cell fusion is particularly sensitive to the C-terminal charge of HAP2(GCS1).

All evidence to date indicates that HAP2(GCS1) has an essential role in fertilization [2]-[4],[7],[9],[15], but its exact function remains unknown. Observations made in Chlamydomonas suggest it is required for gamete fusion because gamete attraction and binding/juxtaposition of membranes are normal in *HAP2(GCS1)* loss-of-function *minus* gametes, yet membranes fail to fuse [7]. One hypothesis is that HAP2(GCS1) directly catalyzes membrane fusion [19]. While HAP2(GCS1) does not share primary sequence with known fusogenic proteins, it shares features with the FAST proteins of non-enveloped viruses. FAST proteins have a single transmembrane domain, a conserved, extracellular N-terminus and a variable C-terminus that is positively charged [18]. A cell expressing FAST proteins can fuse with a non-expressing neighboring cell [18],[20], so like HAP2(GCS1), the requirement for FAST proteins in fusion is asymmetric. Thus, by virtue of their common attributes, HAP2(GCS1) and FAST proteins may use a similar mechanism to catalyze membrane fusion.

We have mapped the key domains of HAP2(GCS1) and propose a model in which the N-terminus functions by interacting with female gamete-expressed proteins and the C-terminus is required to interact with the plasma membrane through its positive charge. By analogy to known fusogenic proteins, we propose that these interactions bring gamete membranes into close proximity, destabilize the phospholipid bilayer, and generate membrane structures favoring their fusion [19], [21]–[23]. Future studies designed to directly assess the ability of HAP2(GCS1) to catalyze membrane fusion will be required to test this model and to elucidate the biochemical function of this ancient reproductive protein.

## Materials and Methods

### Maintenance of Plants

All seeds were plated onto solid Murashige and Skoog (MS) medium (MP Biomedicals LLC, Solon, OH, USA) supplemented with 0.5% sucrose containing 25 µg/mL glufosinate ammonium (Basta; Sigma Aldrich/Riedel-de Haën, St. Louis, MO, USA) and/or 50 µg/mL kanamycin sulfate (Sigma-Aldrich, St. Louis, MO, USA). Seedlings were transplanted to sterile 2MIX potting medium (Conrad Fafard Incorporated, Agawam, MA, USA) and grown at 20°C, 16 day/8 night hour light cycle in a GCW30 walk-in Arabidopsis chamber (Environmental Growth Changes, Chagrin Falls, OH, USA) at 50–60% humidity. Plants were bottom-watered with 0.5X 15-5-15 (N-P-K) Peters Professional fertilizer (The Scotts Company, Marysville, OH, USA) as needed.

### Plasmid Constructs

Chimeric constructs were made using a modified Arabidopsis *HAP2(GCS1)* CDS. Mutations were made in the CDS to eliminate the endogenous EcoRI site at position 71 (‘A’ of the initiating methionine codon of *A thaliana* is position 1), to create a second BamH I site at position 1618 that complements the endogenous BamH I site at 178, and to create a Bmt I site at position 1740. Each directed mutation was made using the QuickChange mutagenesis protocol (Stratagene/Agilent Technologies, Santa Clara, CA, USA). Additional changes and sequence swaps were introduced through linker primers that contain both appropriate restriction sites and new sequences.

All CDS variants were subcloned into a custom vector based on the pTAT backbone [24] that contains sequence encoding tandem V5 [11] and tetra-cysteine (CCGPCC) [12] epitope tags downstream of the multiple cloning site. The tagged CDS was then moved into pCamHap2, a variant of pCambia2300 (Genbank: AF234315; [25], containing ∼1.5 kb of the endogenous Arabidopsis *HAP2(GCS1)* promoter [4] and 19S terminator flanking a modified multiple cloning site (Figure 1).

### Transformation of *hap2-1/+* and Selection of Transgenic Lines

Each recombinant pCamHap2 T-DNA plasmid was transformed into Agrobacterium strain GV3101 [26] and resultant colonies were expanded for floral dipping [27]. T_1_ plants were selected on MS plates containing both Basta and kanamyacin, but subsequent generations were selected on either Basta or kanamyacin (Figure 1). Fifteen to twenty-four T_1_ plants were screened, and at least 2 lines were selected for further analysis with ∼66% Basta^R^ (expected full rescue in T_2_ plants). In cases where complementation failed and no lines resulted in >50% Basta^R^, lines with the highest percentage Basta^R^ were analyzed. Transgenic lines with a single insertion of the CDS construct were selected based on kan^R^ data (expect 75–83% kan^R^).

### GUS Staining

Stage 12 flowers or pistils dissected 7.5 hour after manual pollination were fixed and stained for GUS according to previously published methods [2],[4].

### Quantitative Reverse Transcription Polymerase Chain Reaction

Stage 12–14 flowers were collected, frozen in liquid nitrogen, and stored at −80°C until needed. RNA was isolated from 25–50 flowers per line using Qiagen RNA Mini columns (Qiagen Corporation, Valencia, CA, USA), including the optional on-column DNase treatment. Complementary first strand DNA was synthesized from 1 µg of total RNA with random hexamers and poly dT primers using the TaqMan kit (Applied Biosystems, Foster City, CA, USA). One fortieth of each reverse transcription reaction was used per 25 µL quantitative real-time PCR (qPCR) replicate. qPCR was completed on a 7300 Real-Time PCR System (Applied Biosystems, Foster City, CA, USA) using Platinum Taq SYBR Green Mix with ROX (Invitrogen Corporation, Carlsbad, CA, USA). Amplification was quantified over 40 cycles of 95°C, 0∶15 denaturation and 60°C, 2∶30 extension, and amplicons were evaluated with a standard dissociation step. Primers for the *HAP2(GCS1)* transmembrane domain (F = 5′- TCCAACAAATGCTCGAGTTTC; R = 5′- ATTGGGAAGAGAGCGAGGAG; 101 bp), *H3.3* (At1g19890; F = 5′- ATTGCCTTTCCAACGACTTG; R = 5′- AACAACCCCACCAAGTATGC; 120 bp) and the exogenous V5C4 dual epitope (F = 5′- CCTAACCCTCTCCTCGGTCT; R = 5′- TCCCTTATCGGGAAACTACTCA) were used at a final concentration of 100 nM.

C_t_ values of triplicate reactions were averaged per sample, normalized to sperm-expressed histone *H3.3* transcript [28]–[29], and compared to the expression levels in *hap2-1/+* flowers (transmembrane primers) or presented without fold-change ratios (V5C4 epitope primers). This difference in data presentation is due to the nature of the targets of each primer set: The transmembrane primers (TM) amplify the same sequence from both endogenous *HAP2(GCS1)* and CDS transcripts. This primer set thus allows us to quantify how much more *HAP2(GCS1*) variant mRNA is present compared to *hap2-1/+*. The epitope tag primers (V5C4) only recognize the CDS transgene (Figure S2D), thus only a normalized value may be presented against a similar amplification from plants that lack this tag.

### Embryo Sac Analysis by Chloral Hydrate Clearing

Pistils were dissected 48 (*ms1* crosses) or 96 hours (self-pollinated pistils) after manual pollination, and prepared for analysis by chloral hydrate clearing [30].

### Statistics

The two-tailed Student's *t*-test was used to evaluate differences in transmission of *hap2-1*. Significance was assigned based on *p*-values <10^−5^.

## Supporting Information

Figure S1 Compilation of data for each *HAP2(GCS1)* variant. Schematic of each variant (left) is paired with the data from each transgenic line and is compared to *hap2-1/+* (‡, [2] and ¥, [4]. (A) Schematic of each variant. (B-D) Data presented are: (B) Basta^R^ and kan^R^ among T_2_ progeny resulting from self-fertilization of the primary transformant; (C) percentage of T_3_ progeny with specific *hap2-1* genotypes (see also Figure 1 and Figure 2) based on tetrad scoring; and (D) percentage of Basta^R^ progeny from *ms1* cross with pollen from CDS homozogyous, *hap2-1/+* T_3_ plants (see also Figure 1 and Figure 2).
(0.37 MB TIF)

Figure S2 Transcript levels of *HAP2(GCS1)* variants in *hap2-1/+,* CDS/CDS transgenic lines. (A-C) mRNA abundance of the CDS variants. Quantitative real-time PCR data was normalized to values for sperm-expressed histone H3.3 [28],[29]. The two values indicate the range obtained for each transgenic line, from total RNA extracted from 25 flowers pooled from 5 individuals representing descendents of two individuals from each line. (A) Schematic of each variant. (B) ΔC_t_ value for the V5C4 epitope tag mRNA sequence; values greater than 12, based on additional negative controls (data not shown), indicates an absence of mRNA. Note lower ΔC_t_ values denote the presence of more mRNA in each sample. (C) Relative abundance of each CDS variant, compared to *hap2-1/+*. The quantity of mRNA encoding the transmembrane domain, which is shared by endogenous *HAP2(GCS1)* and all variants, was measured and expressed relative to *hap2-1/+*. Expression levels higher than one correspond to transcript quantities of CDS variants greater than found in *hap2-1/+* flowers. (D) Representative ethidium bromide-stained agarose gel of qPCR amplification of the V5C4 epitope tag from the control line (AtN•AtC) or constructs that failed to complement *hap2-1* (see Figure 1 and Figure 2). Neither a control LAT52:GUS transgenic line (LAT52:GUS) or *hap2-1/+* contain a sequence corresponding to the epitope tag, and are thus negative with a ΔC_t_ value >12.
(0.52 MB TIF)

Figure S3 Alignment of HAP2(GCS1) orthologs used. (A) Schematic of the relationship between mRNA and CDS of *A. thaliana* HAP2(GCS1). Vertical lines in the CDS represent exon:exon junctions, these positions are marked by carets in B. (B) Primary sequence alignment of the N-terminal region for the three HAP2(GCS1) orthologs used in this study in the context of the entire Arabidopsis CDS. Amino acid identity at respective positions in the Arabidopsis sequence is shown with a dot (•); gaps in alignments are shown with a dash (-). Key Arabidopsis amino acid position numbers are given above the sequence.
(0.30 MB TIF)

## References

[1] Swanson WJ, Vacquier VD (2002). The rapid evolution of reproductive proteins.. Nat Rev Genet.

[2] Johnson MA, von Besser K, Zhou Q, Smith E, Aux G (2004). Arabidopsis *hapless* mutations define essential gametophytic functions.. Genetics.

[3] Mori T, Kuroiwa H, Higashiyama T, Kuroiwa T (2006). GENERATIVE CELL SPECIFIC 1 is essential for angiosperm fertilization.. Nat Cell Biol.

[4] von Besser K, Frank AC, Johnson MA, Preuss D (2006). Arabidopsis *HAP2(GCS1)* is a sperm-specific gene required for pollen tube guidance and fertilization.. Development.

[5] Swanson R, Edlund AF, Preuss D (2004). Species specificity in pollen-pistil interactions.. Annu Rev Genet.

[6] Berger F, Hamamura Y, Ingouff M, Higashiyama T (2008). Double fertilization - caught in the act.. Trends Plant Sci.

[7] Liu Y, Tewari R, Ning J, Blagborough AM, Garbom S (2008). The conserved plant sterility gene *HAP2* functions after attachment of fusogenic membranes in *Chlamydomonas* and *Plasmodium* gametes.. Genes Dev.

[8] Steele RE, Dana CE (2009). Evolutionary history of the HAP2/GCS1 gene and sexual reproduction in metazoans.. PLoS ONE.

[9] Hirai M, Arai M, Mori T, Miyagishima SY, Kawai S (2008). Male fertility of malaria parasites is determined by GCS1, a plant-type reproduction factor.. Curr Biol.

[10] Preuss D, Rhee SY, Davis RW (1994). Tetrad analysis possible in Arabidopsis with mutation of the *QUARTET (QRT)* genes.. Science.

[11] Southern JA, Young DF, Heaney F, Baumgartner WK, Randall RE (1991). Identification of an epitope on the P and V proteins of simian virus 5 that distinguishes between two isolates with different biological characteristics.. J Gen Virol.

[12] Adams SR, Campbell RE, Gross LA, Martin BR, Walkup GK (2002). New biarsenical ligands and tetracysteine motifs for protein labeling in vitro and in vivo: synthesis and biological applications.. J Am Chem Soc.

[13] Wolfe KH, Gouy M, Yang YW, Sharp PM, Li WH (1989). Date of the monocot-dicot divergence estimated from chloroplast DNA sequence data.. Proc Natl Acad Sci U S A.

[14] Bailey CD, Koch MA, Mayer M, Mummenhoff K, O'Kane SL (2006). Toward a global phylogeny of the Brassicaceae.. Mol Biol Evol.

[15] Blagborough AM, Sinden RE (2009). Plasmodium berghei HAP2 induces strong malaria transmission-blocking immunity in vivo and in vitro.. Vaccine.

[16] Haygood R (2004). Sexual conflict and protein polymorphism.. Evolution Int J Org Evolution.

[17] Okuda S, Tsutsui H, Shiina K, Sprunck S, Takeuchi H (2009). Defensin-like polypeptide LUREs are pollen tube attractants secreted from synergid cells.. Nature.

[18] Shmulevitz M, Duncan R (2000). A new class of fusion-associated small transmembrane (FAST) proteins encoded by the non-enveloped fusogenic reoviruses.. EMBO J.

[19] Wong JL, Johnson MA (2010). Is HAP2-GCS1 an ancestral gamete fusogen?. Trends Cell Biol Jan 15. [Epub ahead of print].

[20] Corcoran JA, Salsman J, de Antueno R, Touhami A, Jericho MH (2006). The p14 fusion-associated small transmembrane (FAST) protein effects membrane fusion from a subset of membrane microdomains.. J Biol Chem.

[21] Basanez G (2002). Membrane fusion: the process and its energy suppliers.. Cell Mol Life Sci.

[22] Chernomordik LV, Kozlov MM (2008). Mechanics of membrane fusion.. Nat Struct Mol Biol.

[23] Wickner W, Schekman R (2008). Membrane fusion.. Nat Struct Mol Biol.

[24] Nagahara H, Vocero-Akbani AM, Snyder EL, Ho A, Latham DG (1998). Transduction of full-length TAT fusion proteins into mammalian cells: TAT-p27Kip1 induces cell migration.. Nat Med.

[25] Hajdukiewicz P, Svab Z, Maliga P (1994). The small, versatile pPZP family of Agrobacterium binary vectors for plant transformation.. Plant Mol Biol.

[26] Koncz C, Schell J (1986). The Promoter of Tl-DNA Gene 5 Controls the Tissue-Specific Expression of Chimeric Genes Carried by a Novel Type of Agrobacterium Binary Vector.. Molecular & General Genetics.

[27] Clough SJ, Bent AF (1998). Floral dip: a simplified method for Agrobacterium-mediated transformation of Arabidopsis thaliana.. Plant J.

[28] Ingouff M, Hamamura Y, Gourgues M, Higashiyama T, Berger F (2007). Distinct dynamics of HISTONE3 variants between the two fertilization products in plants.. Curr Biol.

[29] Okada T, Endo M, Singh MB, Bhalla PL (2005). Analysis of the histone H3 gene family in Arabidopsis and identification of the male-gamete-specific variant *AtMGH3*.. Plant J.

[30] Yadegari R, Depaiva GR, Laux T, Koltunow AM, Apuya N (1994). Cell-Differentiation and Morphogenesis Are Uncoupled in Arabidopsis *raspberry* Embryos.. Plant Cell.

