# Supplementary figures and images for: HAP2(GCS1)-Dependent Gamete Fusion Requires a Positively Charged Carboxy-Terminal Domain
Source: PLoS Genet. 2010 Mar 19;6(3):e1000882. doi: 10.1371/journal.pgen.1000882 (PMC2841615; doi:10.1371/journal.pgen.1000882)

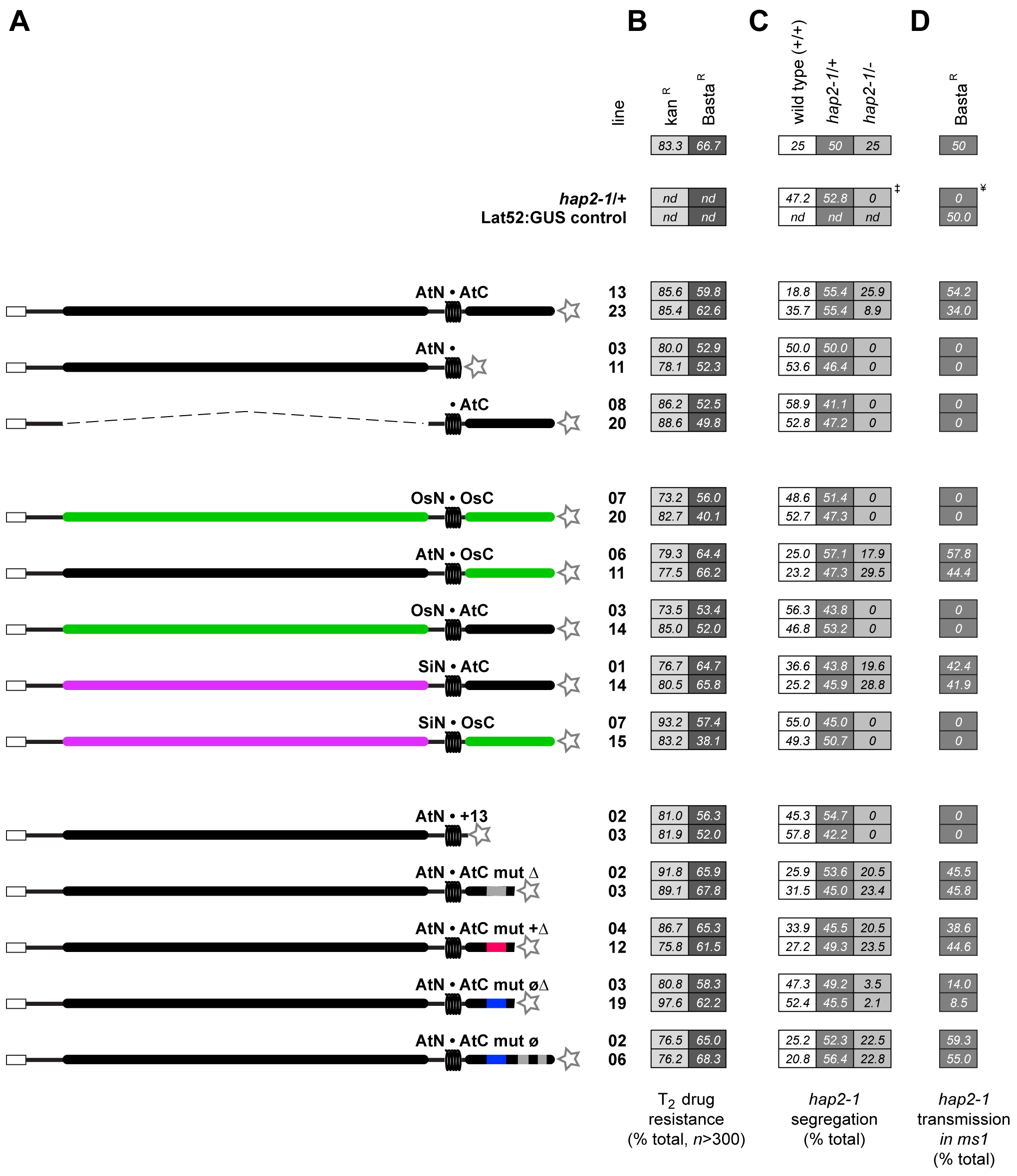

Supplement: Figure S1 — Compilation of data for each HAP2(GCS1) variant. Schematic of each variant (left) is paired with the data from each transgenic line and is compared to hap2-1/+ (‡, [2] and ¥, [4]. (A) Schematic of each variant. (B-D) Data presented are: (B) BastaR and kanR among T2 progeny resulting from self-fertilization of the primary transformant; (C) percentage of T3 progeny with specific hap2-1 genotypes (see also Figure 1 and Figure 2) based on tetrad scoring; and (D) percentage of BastaR progeny from ms1 cross with pollen from CDS homozogyous, hap2-1/+ T3 plants (see also Figure 1 and Figure 2). (0.37 MB TIF) [file pgen.1000882.s001.tif]

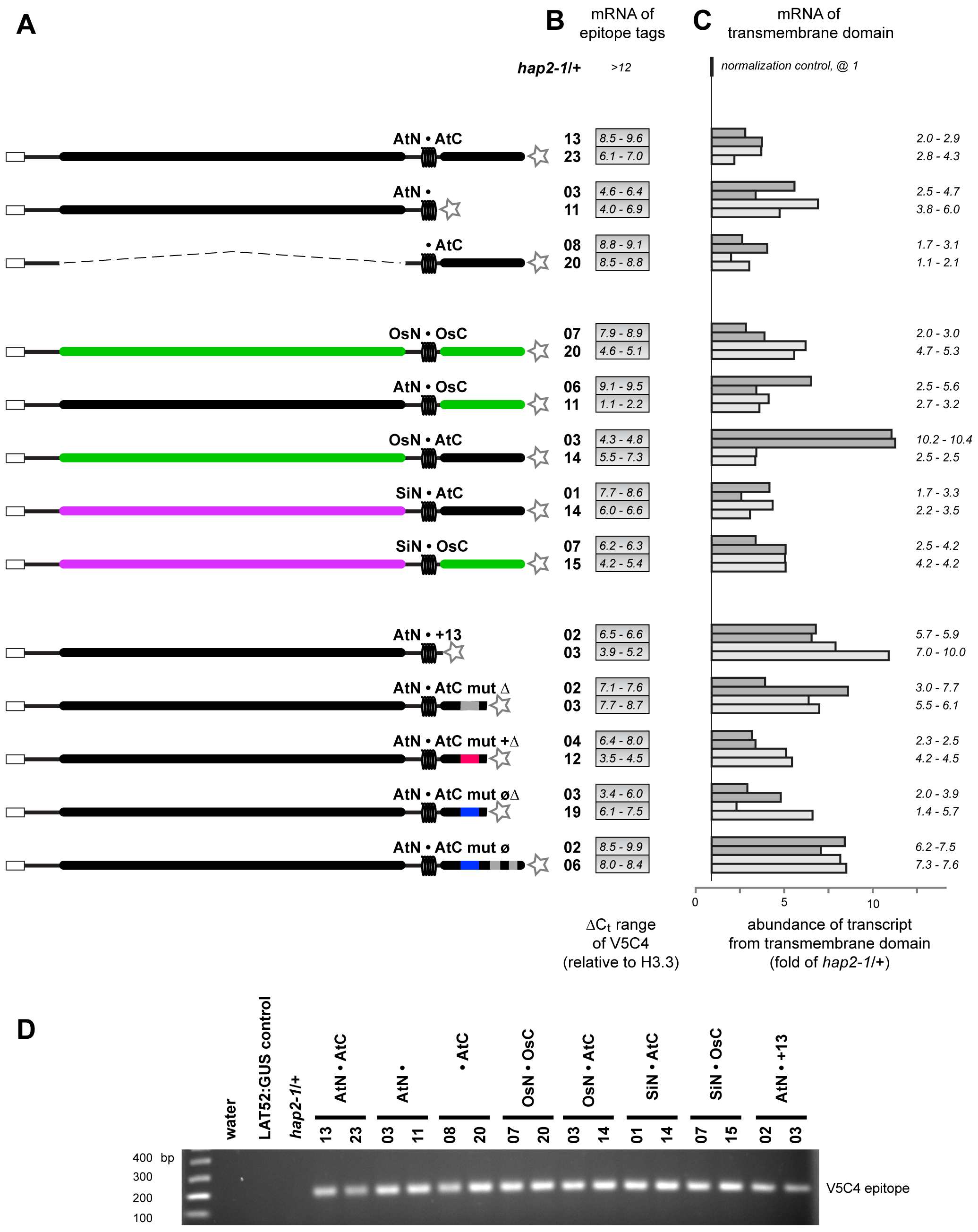

Supplement: Figure S2 — Transcript levels of HAP2(GCS1) variants in hap2-1/+, CDS/CDS transgenic lines. (A-C) mRNA abundance of the CDS variants. Quantitative real-time PCR data was normalized to values for sperm-expressed histone H3.3 [28],[29]. The two values indicate the range obtained for each transgenic line, from total RNA extracted from 25 flowers pooled from 5 individuals representing descendents of two individuals from each line. (A) Schematic of each variant. (B) ΔCt value for the V5C4 epitope tag mRNA sequence; values greater than 12, based on additional negative controls (data not shown), indicates an absence of mRNA. Note lower ΔCt values denote the presence of more mRNA in each sample. (C) Relative abundance of each CDS variant, compared to hap2-1/+. The quantity of mRNA encoding the transmembrane domain, which is shared by endogenous HAP2(GCS1) and all variants, was measured and expressed relative to hap2-1/+. Expression levels higher than one correspond to transcript quantities of CDS variants greater than found in hap2-1/+ flowers. (D) Representative ethidium bromide-stained agarose gel of qPCR amplification of the V5C4 epitope tag from the control line (AtN•AtC) or constructs that failed to complement hap2-1 (see Figure 1 and Figure 2). Neither a control LAT52:GUS transgenic line (LAT52:GUS) or hap2-1/+ contain a sequence corresponding to the epitope tag, and are thus negative with a ΔCt value >12. (0.52 MB TIF) [file pgen.1000882.s002.tif]

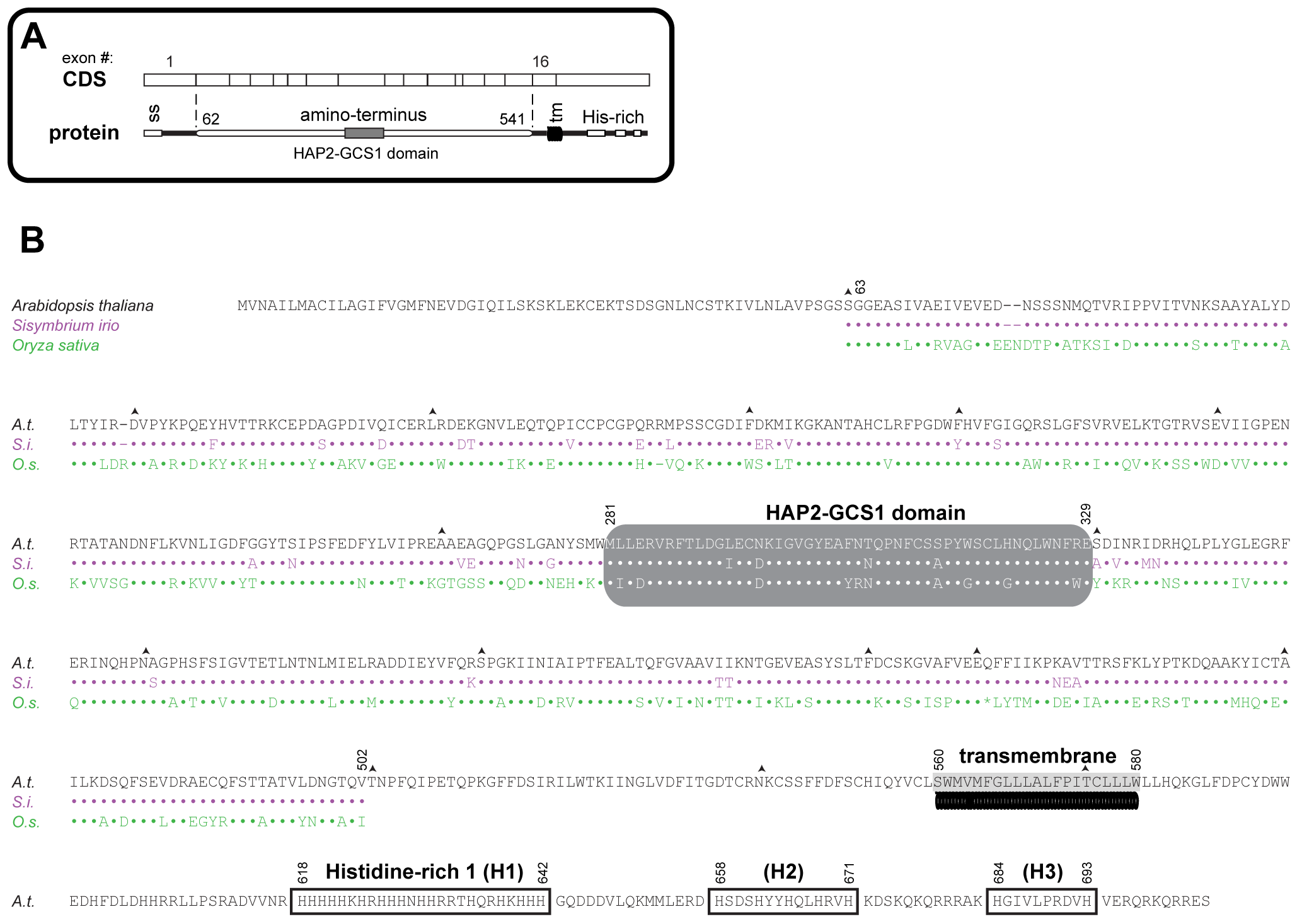

Supplement: Figure S3 — Alignment of HAP2(GCS1) orthologs used. (A) Schematic of the relationship between mRNA and CDS of A. thaliana HAP2(GCS1). Vertical lines in the CDS represent exon:exon junctions, these positions are marked by carets in B. (B) Primary sequence alignment of the N-terminal region for the three HAP2(GCS1) orthologs used in this study in the context of the entire Arabidopsis CDS. Amino acid identity at respective positions in the Arabidopsis sequence is shown with a dot (•); gaps in alignments are shown with a dash (-). Key Arabidopsis amino acid position numbers are given above the sequence. (0.30 MB TIF) [file pgen.1000882.s003.tif]
